# Intrinsic MicroRNA‐10a Restricts Regulatory T Cell Suppressive Function and Intestinal Repair by Coordinating Transcriptional, Metabolic, and Epithelial Repair Pathways

**DOI:** 10.1002/advs.202509953

**Published:** 2025-11-03

**Authors:** Wenjing Yang, Tianming Yu, Hui Yang, Suxia Yao, Ruihua Ma, Elizabeth M Steinert, Karen M Ridge, Weiguo Cui, Navdeep S Chandel, Yingzi Cong

**Affiliations:** ^1^ Division of Gastroenterology and Hepatology Department of Medicine Northwestern University Feinberg School of Medicine Chicago IL 60611 USA; ^2^ Center for Human Immunobiology Northwestern University Feinberg School of Medicine Chicago IL 60611 USA; ^3^ Department of Microbiology and Immunology University of Texas Medical Branch Galveston TX 77555 USA; ^4^ Division of Pulmonary and Critical Care Medicine Department of Medicine Northwestern University Feinberg School of Medicine Chicago IL 60611 USA; ^5^ Department of Pathology Northwestern University Feinberg School of Medicine Chicago IL 60611 USA

**Keywords:** intestinal inflammation, intestinal repair, microRNA, mitochondria, oxidative phosphorylation

## Abstract

Regulatory T cells (Tregs) are indispensable for maintaining immune homeostasis and suppressing excessive inflammatory responses in the intestine. While much attention has focused on positive regulators of Treg differentiation and function, the mechanisms that constrain Treg transcriptional and metabolic programs remain poorly understood. Here, miR‐10a is identified as a key negative regulator of Treg suppressive capacity and their crosstalk with intestinal epithelial cells. Single‐cell and bulk transcriptomic analyses reveal that Treg‐specific deletion of miR‐10a promotes an effector Treg (eTreg) phenotype characterized by elevated Blimp1 expression, a direct target of miR‐10a. MiR‐10a‐deficient Tregs demonstrate enhanced suppressive capacity in alleviating colitis without compromising Treg stability. Mechanistically, miR‐10a deficiency drives metabolic reprogramming, highlighted by altered mitochondrial oxidative phosphorylation through *Uqcrq*, a component of mitochondrial complex III. Loss of *Uqcrq* impairs Treg suppressive function in colitis but does not affect stability. Furthermore, miR‐10a targets amphiregulin (Areg), an epidermal growth factor‐like molecule crucial for mucosal epithelial repair. Areg‐deficient Tregs exhibit decreased intestinal barrier function, whereas miR‐10a‐deficient Tregs exhibit enhanced barrier function in experimental colitis. These findings define a multifaceted role for intrinsic miR‐10a in negatively regulating Treg function by integrating transcriptional, metabolic, and epithelial repair pathways, thereby unveiling potential therapeutic interventions in inflammatory bowel diseases.

## Graphical abstract

This study identifies microRNA‐10a (miR‐10a) as a key brake on regulatory T cell (Treg) suppressive function and intestinal repair. By targeting Blimp‐1, Uqcrq, and amphiregulin, miR‐10a restrains transcriptional, metabolic, and epithelial programs essential for Treg activity. Loss of miR‐10a enhances Treg suppression of colitis and promotes mucosal healing, revealing therapeutic potential for inflammatory bowel disease.

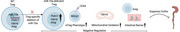

## 1 Main

The essential role of Regulatory T cells (Tregs) has been well‐established for regulating immune responses, maintaining tolerance to self‐antigens, and preventing autoimmune inflammatory diseases and associated pathologies.^[^ ^1^, ^2^ ^]^ Tregs can inhibit effector T cell proliferation and cytokine production by producing TGFβ and IL‐10 and thereby play a vital role in limiting immune‐mediated inflammation.^[^ ^2^, ^3^ ^]^ Tregs can also promote mucosal tissue repairs by producing amphiregulin (Areg),^[^ ^4^ ^]^ an epidermal growth factor‐like molecule. Tregs are defined by a core regulatory program established by the lineage‐specifying transcription factor Foxp3, which is essential for their differentiation and function. Multiple mechanisms have been identified to drive Foxp3^+^ Treg differentiation, function, and maintain identity.^[^ ^5^ ^]^ The maintenance of the Treg program requires other transcription factors that act as genetic modulators. These transcription factors can either be supportive of Foxp3^[^ ^6^ ^]^ or independent and additive to Foxp3.^[^ ^7^ ^]^ Under certain contexts, the transcription factors Blimp1 and Irf4 can synergize with Foxp3 to trigger a reinforced effector Treg program.^[^ ^8^ ^]^ Emerging evidence indicates that inflammatory Tregs and functional effector Tregs (eTregs) are present in inflamed tissues in autoimmune inflammatory diseases, such as inflammatory bowel diseases (IBD).^[^ ^9^ ^]^ Although whether Treg can function and restrain the progress of severe autoimmune inflammatory diseases under inflammatory conditions is still debatable, accumulating evidence in experimental animal models demonstrates that proinflammatory cytokines, such as interleukin (IL)‐1, IL‐4, IL‐6, and IL‐23, can drive Tregs to lose Foxp3 expression and acquire proinflammatory features, and become functionally impaired.^[^ ^10^, ^11^ ^]^

T cell differentiation and activation status are intricately linked to their metabolic signatures.^[^ ^12^, ^13^ ^]^ Upon activation, naive T cells switch their metabolic program from oxidative phosphorylation (OXPHOS) to aerobic glycolysis and become rapidly dividing effector T cells.^[^ ^13^ ^]^ However, Tregs exhibit a unique metabolic profile and rely predominantly on an increase in mitochondrial metabolism to maintain their suppressive phenotype, further promoted by the expression of Foxp3.^[^ ^14^ ^]^ It has been shown that the mitochondrial respiratory chain is required for Treg suppression capacity.^[^ ^15^ ^]^ Despite the intensive effort and great achievement in investigating the positive regulators for Treg differentiation and function, the negative regulators that constrain Treg transcriptional and metabolic programs to restrict Treg differentiation and function remain poorly understood. Emerging evidence indicates that microRNAs (miRNAs), a class of short noncoding RNAs and epigenetic regulators, play a crucial role in regulating Treg differentiation and function. Dicer‐deficient Tregs are unstable and express lower levels of Foxp3 and altered levels of multiple genes associated with Treg function.^[^ ^16^ ^]^ Treg expression of miR‐146a is critical for their suppression function, and miR‐146a deficiency in Tregs leads to severe inflammation in multiple organs.^[^ ^17^ ^]^ MiR‐31 and miR‐21 differentially regulate human Treg Foxp3 expression.^[^ ^18^ ^]^ miR‐10a is highly expressed in Tregs.^[^ ^19^ ^]^ It has been shown that miR‐10a controls the conversion of induced Tregs into T follicular helper (Tfh) cells by targeting the expression of the transcriptional repressor Bcl‐6 and the corepressor Ncor2.^[^ ^20^ ^]^ However, whether and how miR‐10a regulates Treg function is still unknown.

In this report, we demonstrate that miR‐10a negatively regulates Treg function but does not affect Treg stability and differentiation. Treg‐specific miR‐10a knock‐out Foxp3^YFP‐cre^miR‐10a^fl/fl^ mice develop less severe intestinal inflammation upon inflammatory insults, and miR‐10a‐deficient Tregs demonstrate more potent suppressive capacity in inhibiting effector T cells and colitis development. Mechanistically, miR‐10a suppresses mitochondrial OXPHOS by targeting *Uqcrq*, which encodes the QPC subunit of complex III, leading to restricted Treg function. Furthermore, miR‐10a inhibits Treg suppressive function by targeting *Prdm1* and impairs Treg tissue repair function by targeting *Areg*.

## 2 Results

### 2.1 MiR‐10a‐Deficient Treg Demonstrated a More Potent Suppressive Capacity in Inhibiting Colitis

To explore the specific role of miR‐10a in Tregs, we generated Treg‐specific miR‐10a‐deficient Foxp3^YFP‐cre^miR‐10a^fl/fl^ mice. There were no significant differences in major immune cell populations, including CD4^+^ T cells, CD8^+^ T cells, B220^+^ B cells, CD11c^+^ dendritic cells, macrophages, and neutrophils, in the spleens and intestines between Foxp3^YFP‐cre^miR‐10a^fl/fl^ mice and WT Foxp3^YFP‐cre^miR‐10a^fl/+^ mice under steady conditions (Figure S1A–T, Supporting Information).

**Figure 1:**
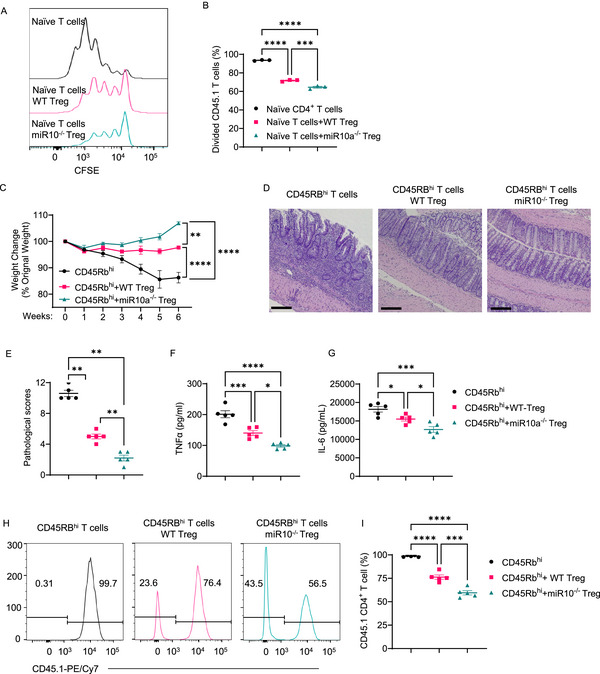
MiR‐10a‐deficient Tregs display a more potent suppressive activity. A, B) CFSE‐labeled CD45.1^+^ naïve CD4^+^ T cells were co‐cultured with or without CD45.2^+^ WT or miR‐10a‐deficient Tregs (*n* = 3 per group) in the presence of irradiated antigen‐presenting cells and soluble anti‐CD3 antibody for 60 h. Representative flow cytometry histogram plots of CFSE intensity in CD45.1^+^ CD4^+^ T cells (A). Quantification of divided CD45.1^+^ CD4^+^ T cells (B). C–I) Splenic CD45.1^+^ CD4^+^ CD45Rb^hi^ were transferred to *Rag1* ^−/−^ mice together with or without CD45.2^+^ WT or miR‐10a‐deficient Tregs. Recipients (*n* = 5 per group) were sacrificed 6 weeks later. Weight changes of recipient mice (C). Representative H&E (D) and Pathological scores (E) of recipient mice. Colonic secretion of TNF‐α (F) and IL‐6 (G) from recipients. Representative flow cytometry histogram plots (H) and quantification (I) of colonic CD45.1^+^ cells in CD4^+^ T cells. Scale bar, 100 µm. All data are presented as mean ± SEM and are one representative of three independent experiments (A–I). One‐way ANOVA with Tukey's multiple comparisons test (B, F, G, and I), Repeated Measures Two‐way ANOVA with Sidak's multiple comparisons test (C), Mann–Whitney U test (E); ^*^ *p* < 0.05, ^**^ *p* < 0.01, ^***^ *p* < 0.001, ^****^ *p* < 0.0001.

To investigate whether miR‐10a affects Treg suppressive function on T cells, we purified the splenic miR‐10a‐deficient and WT Foxp3^+^ Tregs from Foxp3^YFP‐cre^miR‐10a^fl/fl^ mice and WT Foxp3^YFP‐cre^miR‐10a^fl/+^ mice, respectively, and co‐cultured them with CFSE‐labeled CD45.1 naϊve CD4^+^ T cells in the presence of anti‐CD3 antibody and irradiated antigen‐presenting cells. We found that while both WT and miR‐10a‐deficient Tregs suppressed CD4^+^ T cell proliferation, CD4^+^ T cells co‐cultured with miR‐10a‐deficient Tregs divided less than those co‐cultured with WT Tregs (**Figure** **1**A,B), indicating that miR‐10a‐deficient Tregs exhibit more potent suppressive function.

To investigate the role of miR‐10a‐deficient Tregs in inhibiting intestinal inflammation in vivo, we isolated CD45.1 CD4^+^ CD45Rb^hi^ T cells and intravenously transferred them into immune‐deficient *Rag1* ^−/−^ mice together with or without WT or miR‐10a‐deficient Tregs at the ratio of 2:1. At this ratio, Tregs do not completely inhibit colitis. The weight change was monitored weekly. The mice were sacrificed six weeks after cell transfer, and the severity of colitis was assessed by histopathology and intestinal proinflammatory cytokine production. *Rag1* ^−/−^ mice receiving CD4^+^ CD45Rb^hi^ T cells alone developed severe colitis, as evidenced by increased weight loss (Figure 1C), higher pathological scores (Figure 1D,E), and intestinal TNF‐α and IL‐6 levels (Figure 1F,G), which were inhibited by co‐transfer of WT or miR‐10a‐deficient Tregs. Interestingly, miR‐10a‐deficient Tregs showed more potent suppressive capacity in inhibiting colitis development than WT Tregs (Figure 1C‐G). Additionally, the percentage of intestinal CD45.1^+^ CD4^+^ T cells in total CD4^+^ T cells was lower in recipients of CD4^+^ CD45Rb^hi^ T cells with miR‐10a‐deficient Tregs compared to *Rag1* ^−/−^ recipients of CD4^+^ CD45Rb^hi^ T cells with WT Tregs (Figure 1H,I), indicating that miR‐10a‐deficient Tregs exhibit more potent inhibition of T cell proliferation in vivo compared to WT Tregs.

To confirm the role of Treg‐expressed miR‐10a in regulating intestinal inflammation, we used the DSS‐induced chronic colitis model, which is more physiologically relevant to human colitis. We subjected WT Foxp3^YFP‐cre^miR‐10a^fl/+^ mice and Foxp3^YFP‐cre^miR‐10a^fl/fl^ mice to 3 cycles of 2% DSS in drinking water for 7 days, followed by 7 days of regular drinking water. At the end of the experiment, the mice were sacrificed to assess the severity of colitis by analyzing histopathological findings. Foxp3^YFP‐cre^miR‐10a^fl/fl^ mice demonstrated less intestinal inflammation than WT Foxp3^YFP‐cre^miR‐10a^fl/+^ mice upon chronic DSS insults (Figure S2A,B, Supporting Information). These data suggested that miR‐10a negatively regulates Treg suppressive function in inhibiting intestinal inflammation.

### 2.2 Intrinsic MiR‐10a Deficiency in Treg Does Not Affect Treg Stability and Differentiation

**Figure 2:**
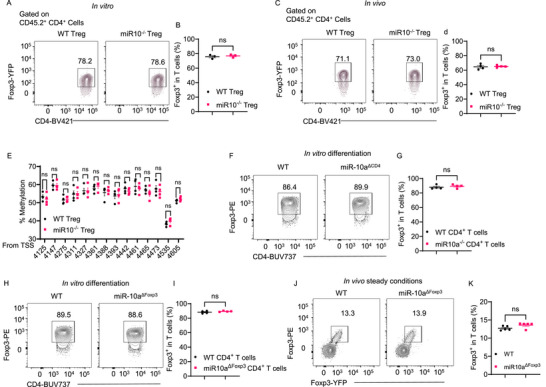
Loss of miR‐10a does not affect Treg stability and differentiation. A, B) CFSE‐labeled CD45.1^+^ naïve CD4^+^ T cells were co‐cultured with or without CD45.2^+^ WT or miR‐10a‐deficient Tregs (*n* = 3) in the presence of irradiated antigen‐presenting cells and soluble anti‐CD3 antibody for 60 h. Representative flow cytometry plots of Foxp3‐YFP^+^ in CD45.2^+^ CD4^+^ T cells (A). Quantification of Foxp3‐YFP^+^ in CD45.2^+^ CD4^+^ T cells (B). C, D) Splenic CD45.1^+^ CD4^+^ CD45Rb^hi^ T cells were transferred to *Rag1* ^−/−^ mice together with or without CD45.2^+^ WT/miR‐10a‐deficient Tregs. Recipients (*n* = 4) were sacrificed 6 weeks later. Representative flow cytometry plots (C) and quantification (D) of colonic Foxp3‐YFP^+^ in CD45.2^+^ CD4^+^ T cells. E) Splenic Tregs were isolated from WT and miR‐10a‐deficient Tregs (*n* = 4 per group), and the methylation levels in Treg‐specific demethylated region (TSDR) sites were shown. F, G) Splenic WT and miR‐10‐deficient CD4^+^ T cells (*n* = 4 per group) were activated and cultured under Treg conditions for 5 days. Representative flow cytometry plots (F) and quantification (G) of Foxp3^+^ in CD4^+^ T cells. H, I) Splenic CD4^+^ T cells were isolated from Foxp3^YFP‐cre^miR‐10a^fl/fl^ mice and WT Foxp3^YFP‐cre^miR‐10a^fl/+^ mice (*n* = 4 per group) and cultured under Treg conditions for 5 days. Representative flow cytometry plots (F) and quantification (G) of Foxp3^+^ in CD4^+^ T cells. J, K) Representative flow cytometry plots (J) and quantification (K) of colonic Foxp3^+^ in CD4^+^ T cells from Foxp3^YFP‐cre^miR‐10a^fl/fl^ mice and WT Foxp3^YFP‐cre^miR‐10a^fl/+^ mice (*n* = 5 per group) under steady conditions. All data are presented as mean ± SEM and are one representative of three independent experiments (a–d and f–k). Unpaired Student's *t*‐test (B, D, E, G, I, and K); ns, not significant.

Next, we investigated whether miR‐10a negatively regulates Treg suppressive activity by affecting Treg stability. We co‐cultured CD45.1 naϊve CD4^+^ T cells with CD45.2^+^ WT and miR‐10a‐deficient Foxp3^+^ Tregs in the presence of anti‐CD3 antibody and irradiated antigen‐presenting cells for 5 days. Although miR‐10a‐deficient Foxp3^+^ Tregs were more potent in suppressing CD45.1 CD4^+^ T cell proliferation, as shown in Figure 1A,B, the percentage of Foxp3^+^ cells in CD45.2^+^ populations was similar in miR‐10a‐deficient and WT Foxp3^+^ Tregs (**Figure** 2A,B). In addition, we assessed the Foxp3 expression in miR‐10a‐deficient and WT Foxp3^+^ Tregs in the intestine of *Rag1* ^−/−^ recipients of CD45.1 CD4^+^ CD45Rb^hi^ T cells with CD45.2 WT or miR‐10a‐deficient Tregs six weeks after cell transfer, as described in Figure 1C. We found no difference in Foxp3^+^ cells in CD45.2^+^ populations between miR‐10a‐deficient and WT Foxp3^+^ Tregs in the intestines of recipients (Figure 2C,D). Furthermore, we determined the methylation level of Treg‐specific demethylated region (TSDR),^[^ ^21^ ^]^ indicative of Treg stability, in WT and miR‐10a‐deficient Treg. There was no difference in TSDR methylation between WT and miR‐10‐deficient Tregs (Figure 2E). These data suggested that miR‐10a negatively regulates Treg suppressive function without affecting Treg stability.

To investigate whether miR‐10a in T cells or Treg affects Treg differentiation, we cultured naϊve CD4^+^ T cells isolated from WT CD4^cre^ miR‐10a^fl/+^ mice or CD4^+^ T cell‐specific miR‐10a‐deficient CD4^cre^miR‐10a^fl/fl^ mice under Treg differentiation conditions with TGFβ. There was no difference in Foxp3^+^ Treg differentiation between WT and miR‐10a‐deficient CD4^+^ T cells (Figure 2F,G). We then cultured naϊve CD4^+^ T cells isolated from WT or Treg‐specific miR‐10a‐deficient mice under Treg polarization conditions with TGFβ. Naϊve CD4^+^ T cells from Treg‐specific miR‐10a‐deficient mice demonstrated a similar level of Foxp3^+^ Treg population compared with that from WT mice (Figure 2H,I). Furthermore, we analyzed the intestinal Treg population in Foxp3^YFP‐cre^miR‐10a^fl/fl^ mice and WT mice. We found no difference in the Treg population in the intestines between the two groups of mice (Figure 2J,K). Taken together, those data demonstrated that intrinsic miR‐10a in T cells or Treg does not affect Treg differentiation.

### 2.3 MiR‐10a Deficiency in Treg Promotes Effector Treg and Related Gene Expression

**Figure 3:**
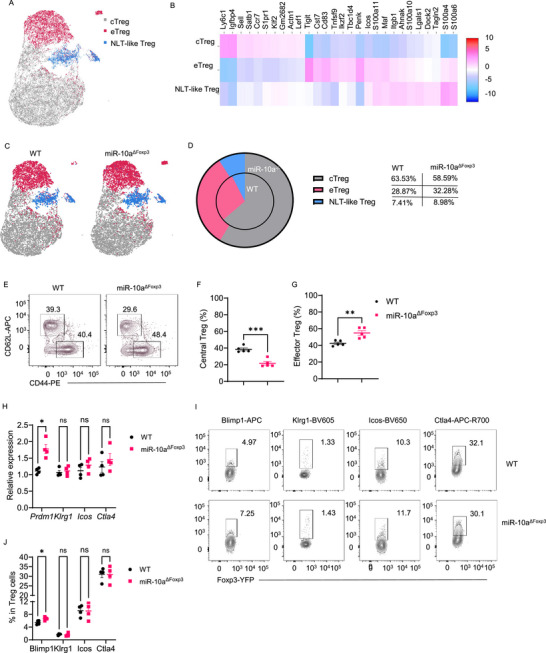
MiR‐10a deficiency in Tregs promotes effector Treg and related gene expression. A–D) Subpopulations and gene expressions in WT and miR‐10a‐deficient Tregs were analyzed by single‐cell RNA sequencing. Cluster visualization in Umap (A) and gene heatmap (B) of subpopulations in all these Tregs. Cluster visualization in Umap (C) and quantification of each cluster (D) in WT and miR‐10a‐deficient Tregs. E–G) Representative flow cytometry plots (E) and quantification (F, G) of central and effector Tregs in WT and miR‐10a‐deficient Tregs (*n* = 5 per group). H) mRNA expression levels of *Prdm1*, *Klrg1*, *Icos*, and *Ctla4* in WT and miR‐10a‐deficient Tregs (*n* = 4 per group). I, J) Representative flow cytometry plots (I) and quantification (J) of Blimp1, Klrg1, Icos, and Ctla4 in WT and miR‐10a‐deficient Tregs (*n* = 4 per group). All data are presented as mean ± SEM and are one representative of two independent experiments (E‐J). Unpaired Student's *t*‐test (F–H, and J); ns, not significant; ^*^ *p* < 0.05, ^**^ *p* < 0.01, ^***^ *p* < 0.001.

We next investigated whether miR‐10a affects Treg subpopulations. We purified Foxp‐YFP^+^ Tregs from the spleens of WT and Treg‐specific miR‐10a‐6deficient mice and analyzed Treg subpopulations by single‐cell RNA sequencing. After quality control filtering, 17,567 cells were retained, with 8264 cells from WT Tregs and 9303 cells from miR‐10a‐deficient Tregs. According to different gene markers, Tregs were clustered into three distinct subpopulations,^[^ ^22^ ^]^ including central Treg (cTreg), effector Treg (eTreg), and non‐lymphoid tissue (NLT)‐like Treg (**Figure** **3**A). cTreg cells are relatively quiescent in the lymphoid tissues, eTreg cells are a more functional subpopulation, and NLT‐like Tregs express eTreg markers and genes related to NLT T cells and tissue homing and trafficking.^[^ ^22^ ^]^ cTreg highly expressed their associated genes, such as *Sell*, *Ly6c1*, *S1pr1*, and *Lgfbp4*, and eTreg expressed their characterized genes, like *Icos*, *Tnfrsf9*, *Ctla4*, and *Tigit* (Figure 3B). Similar to the previous report,^[^ ^22^ ^]^ we detected a less frequent population, named NLT‐like Treg, which expressed some markers other than in eTreg markers, such as *Maf*, *Itgb1*, *Ahnak*, and *Cxcr3* (Figure 3B). Interestingly, miR‐10a‐deficient Treg showed decreased tendency of cTreg and increased tendency of eTreg and NLT‐like eTreg (Figure 3C,D). We confirmed this result by comparing CD62L and CD44 expression in WT and miR‐10a‐deficient Tregs. MiR‐10a‐deficient Tregs showed a lower level of CD62L but a higher expression of CD44 (Figure 3E–G). These data suggested that a deficiency of miR‐10a in Treg leads to an increase in functional eTreg.

We then determined the expression of eTreg‐associated genes in WT and miR‐10a‐deficient Tregs. *Prdm1*, which encodes Blimp1 and is an important eTreg marker,^[^ ^23^ ^]^ was significantly increased in miR‐10a‐deficient Treg compared to WT Tregs (Figure 3H). Consistently, Blimp1 protein expression was increased in miR‐10a‐deficient Tregs detected by flow cytometry (Figure 3I,J). There was no difference in *Klrg1*, *Icos*, and *Ctla4* expression (Figure 3H–J). Furthermore, miR‐10a‐deficient Tregs showed a higher percentage of the *Prdm1* ^+^ population when analyzed in the single‐cell RNA sequencing data (Figure S3, Supporting Information). Additionally, we analyzed bulk RNA‐sequencing to compare the transcriptome of WT and miR‐10a‐deficient Treg and examined other suppressive molecules, including *Cd274* (PDL1), *Il10*, *Il12a* (IL‐35 subunit‐1), Ebi3 (IL‐35 subunit‐2), *Tgfb1*, and *Il2ra* (CD25). Among these, a trend toward increased *Il10* was found in miR‐10a^−/−^ Tregs compared to WT Tregs, while there was no difference in other genes between WT and miR‐10a‐deficient Tregs (Figure S4A,B, Supporting Information).

### 2.4 MiR‐10a Negatively Regulates Treg Suppressive Function by Directly Binding to *Prdm1*

**Figure 4:**
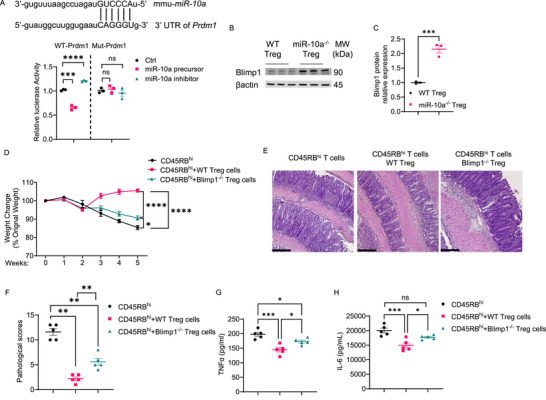
Loss of Blimp1, a downstream target of miR‐10, reduced Treg's capacity to suppress colitis. A) Luciferase activity of *Prdm1* wild‐type (WT) and mutant (Mut) 3′ UTR constructs was measured (*n* = 3 per group). B, C) Representative Western blots showing the expression of Blimp1 and β‐actin in WT and miR‐10a‐deficient Tregs (*n* = 3 per group) (B). Blimp1 protein levels were normalized to β‐actin (*n* = 3 per group) (C). D–H) Splenic CD45.1^+^ CD4^+^ CD45Rb^hi^ were transferred to *Rag1* ^−/−^ mice together with or without CD45.2^+^ WT or Blimp1‐deficient Tregs. Recipients (*n* = 5 per group) were sacrificed 5 weeks later. Weight changes of recipient mice (D). Representative H&E (E) and Pathological scores (F) of recipient mice. Colonic secretion of TNF‐α (G) and IL‐6 (H) from recipients. Scale bar, 100 µm. All data are presented as mean ± SEM and are one representative of two independent experiments (A–H). One‐way ANOVA with Dunnett's multiple comparison test (A), Unpaired Student's *t*‐test (C), Repeated Measures Two‐way ANOVA with Sidak's multiple comparisons test (D), One‐way Mann–Whitney U test (F), ANOVA with Tukey's multiple comparisons test (G, H); ns, not significant; ^*^ *p* < 0.05, ^**^ *p* < 0.01, ^***^ *p* < 0.001, ^****^ *p* < 0.0001.

Interestingly, *Prdm1* is one of the potential targets of miR‐10a predicted by using miRmap, an open‐source software library using thermodynamic, evolutionary, probabilistic, and sequence‐based features.^[^ ^24^ ^]^ We performed a dual luciferase assay to determine whether miR‐10a directly binds to *Prdm1*. We found that miR‐10a precursor decreased, while miR‐10a inhibitor increased, the luciferase activity in a vector containing predicted WT, but not mutant, sequence in the 3′‐UTR of *Prdm1* (**Figure** **4**A). As shown in Figures 3H–J and 4B,C, Blimp1 mRNA and protein expression were increased in miR‐10a‐deficient Treg. These data suggested that miR‐10a suppresses Blimp1 by directly binding to *Prdm1*.

Blimp1 has been reported to be critical in regulating Treg function.^[^ ^8^, ^25^ ^]^ To confirm the impact of Blimp1 on Treg suppressive functions, we isolated Blimp1‐deficient and WT Foxp3^+^ Tregs from Foxp3^YFP‐cre^Blimp1^fl/fl^ mice and Foxp3^YFP‐cre^Blimp1^fl/+^ mice and transferred them into *Rag1* ^−/−^ mice together with CD4^+^ CD45Rb^hi^ T cells. Mouse weights were monitored weekly (Figure 4D). After five weeks, *Rag1* ^−/−^ mice reconstituted with CD4^+^ CD45Rb^hi^ T cells developed severe colitis, which was inhibited by the co‐transfer of WT Tregs. However, Blimp1‐deficient Tregs only partially inhibited colitis (Figure 4D–H). Although the recipient *Rag1* ^−/−^ mice reconstituted with CD4^+^ CD45Rb^hi^ T cells and Blimp1‐deficient Tregs developed less severe colitis compared with that reconstituted with CD4^+^ CD45Rb^hi^ T cells alone, they displayed more severe colitis compared to the mice receiving CD4^+^ CD45Rb^hi^ T cells and WT Tregs, as evidenced by increased weight loss, higher pathological scores, and elevated levels of intestinal TNFα and IL‐6 (Figure 4D–H). These data indicated that miR‐10a controls the Treg suppressive function partially by inhibiting Blimp1 expression.

### 2.5 MiR‐10a Modulates Mitochondrial Metabolism to Negatively Regulate Treg Suppressive Function

**Figure 5:**
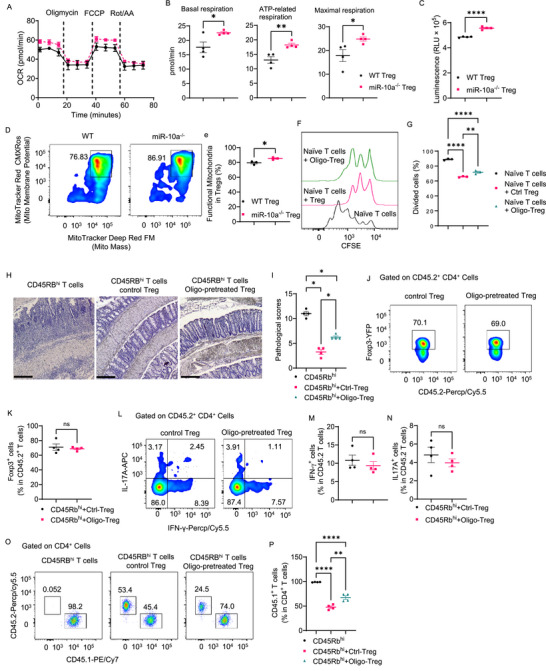
MiR‐10a modulates mitochondrial oxidation in Tregs. A, B) The OCR profile (A) and basal respiration/ATP‐related respiration/maximum respiration (B) in WT and miR‐10a‐deficient Tregs (*n* = 4 per group) were measured by the Mito stress test kit. C) Cellular ATP levels in WT and miR‐10a‐deficient Tregs were shown in a luminescence assay (*n* = 3 per group). D, E) Representative flow cytometry plots (D) and quantification (E) of mitochondrial mass and potential in WT and miR‐10a‐deficient Tregs (*n* = 3 per group). F, G) CFSE‐labeled CD45.1^+^ naïve CD4^+^ T cells were co‐cultured with or without CD45.2^+^ control or Oligomycin‐pretreated Tregs (2 h of pretreatment, *n* = 3 per group) in the presence of irradiated antigen‐presenting cells and soluble anti‐CD3 antibody for 60 h. Representative flow cytometry histogram plots of CFSE intensity in CD45.1^+^ CD4^+^ T cells (F). Quantification of divided CD45.1^+^ CD4^+^ T cells (G). H–P) Splenic CD45.1^+^ CD4^+^ CD45Rb^hi^ were transferred to *Rag1* ^−/−^ mice together with or without CD45.2^+^ control or Oligomycin‐pretreated Tregs. Recipients (*n* = 4 per group) were sacrificed 6 weeks later. Representative H&E (H) and Pathological scores (I) of recipient mice. Representative flow cytometry plots (J) and quantification (K) of colonic Foxp3‐YFP^+^ in CD45.2^+^ CD4^+^ T cells. Representative flow cytometry histogram plots (L) and quantification (M‐N) of IFN‐γ^+^ cells and IL‐17A^+^ cells in CD45.2^+^ CD4^+^ T cells. Representative flow cytometry histogram plots (O) and quantification (P) of colonic CD45.1^+^ cells in CD4^+^ T cells. Scale bar, 100 µm. All data are presented as mean ± SEM and are one representative of two independent experiments (A–P). Unpaired Student's *t*‐test (B, C, E, K, M, and N); one‐way ANOVA with Tukey's multiple comparisons test (G, P), Mann–Whitney U test (I); ns, not significant; ^*^ *p* < 0.05, ^**^ *p* < 0.01, ^****^ *p* < 0.0001.

To further determine how miR‐10a regulates Treg function, we performed genome‐wide RNA‐seq analysis to compare the transcriptome of WT and miR‐10a‐deficient Treg. WT and miR‐10‐deficient Tregs displayed distinct PCA profiles and gene expressions (Figure S4A, Supporting Information). MiR‐10a‐deficient Tregs demonstrated different gene‐enriched pathways and functions, in which cell metabolism is the top Gene Ontology biological process (Figure S4C, Supporting Information). Protein‐protein interaction MCODE network analysis was used to identify neighborhoods where proteins are densely connected. We found that oxidative phosphorylation, one of the top three MCODE networks, was higher in miR‐10a‐deficient Tregs (Figure S4D, Supporting Information). To verify the effect of miR‐10a on oxidative phosphorylation, we determined key parameters of mitochondrial function by using the Mito Stress Kit. A deficiency of miR‐10a led to a higher level of oxygen consumption, including basal respiration, ATP‐related respiration, and maximal respiration (**Figure** **5**A,B). We also determined intracellular ATP levels in miR‐10a‐deficient Tregs. As shown in Figure 5C, miR‐10a^−/−^ Treg showed a higher level of intracellular ATP, supporting enhanced mitochondrial function. Furthermore, we measured the mitochondrial mass and potential between WT and miR‐10a‐deficient Tregs by flow cytometry. Deficiency of miR‐10a increased mitochondrial membrane potential in Tregs (Figure 5D,E). In addition, miR‐10a‐deficient Tregs showed a higher level of mitochondrial reactive oxygen species (ROS) (Figure S4E–G, Supporting Information), a byproduct of oxidative phosphorylation. All these data indicated that miR‐10a negatively regulates mitochondrial oxidation in Tregs.

To determine the role of mitochondrial respiration in Treg function, we pretreated CD45.2 Tregs with 3 nm of oligomycin, an ATP inhibitor that reduces mitochondrial respiration,^[^ ^26^ ^]^ or 100 nm of MitoQ, a mitochondrially targeted antioxidant reducing mitochondrial ROS,^[^ ^27^ ^]^ and co‐cultured them with CSFE‐labeled CD45.1 naïve CD4 T cells. CD45.2 Tregs pretreated with carrier alone served as controls. Pretreatment with oligomycin impaired Treg function in suppressing T cell proliferation in vitro (Figure 5F,G). In contrast, pretreatment with MitoQ enhanced Treg suppressive function (Figure S4H,I, Supporting Information). Oligomycin at 3 nM did not affect Treg apoptosis and proliferation (Figure S5A,B, Supporting Information). To determine whether inhibition of mitochondrial respiration with oligomycin affects Treg function in vivo, we transferred control Tregs or oligomycin‐pretreated Tregs into *Rag1* ^−/−^ mice, together with CFSE‐labeled CBir1 transgenic T cells, which are specific for an immunodominant microbiota antigen.^[^ ^28^ ^]^ The *Rag1* ^−/−^ recipients were sacrificed five days later. We found that the suppressive capability of Tregs pretreated with oligomycin was decreased compared with control Tregs in inhibiting the proliferation of microbiota‐specific T cells (Figure S5C,D, Supporting Information). Furthermore, we determined if the suppressive capability of oligomycin‐pretreated Tregs is impaired in suppressing colitis development. We transferred control Tregs or oligomycin‐pretreated Tregs into *Rag1* ^−/−^ mice, together with CD45Rb^hi^ T cells, and the recipient mice were sacrificed six weeks after cell transfer. While control Tregs inhibited colitis development, the suppressive potential of oligomycin‐pretreated Tregs was impaired, as the *Rag1* ^−/−^ recipients of CD45Rb^hi^ T cells and oligomycin‐pretreated Tregs showed more severe colitis compared to *Rag1* ^−/−^ recipients of CD45Rb^hi^ T cells and control Tregs (Figure 5H,I). Although oligomycin‐pretreated Tregs showed similar Foxp3 expression (Figure 5J,K) and transformed to similar levels of IFN‐γ‐producing Th1 and IL‐17A‐producing Th17 cells (Figure 5I–N) in the intestine of recipient mice after six weeks post‐cell transfer, the capacity of suppressing T cell proliferation was decreased in oligomycin‐treated Tregs compared to control Tregs (Figure 5O,P).

### 2.6 Uqcrq, A Target of miR‐10a, is Indispensable for Treg Suppressive Function

**Figure 6:**
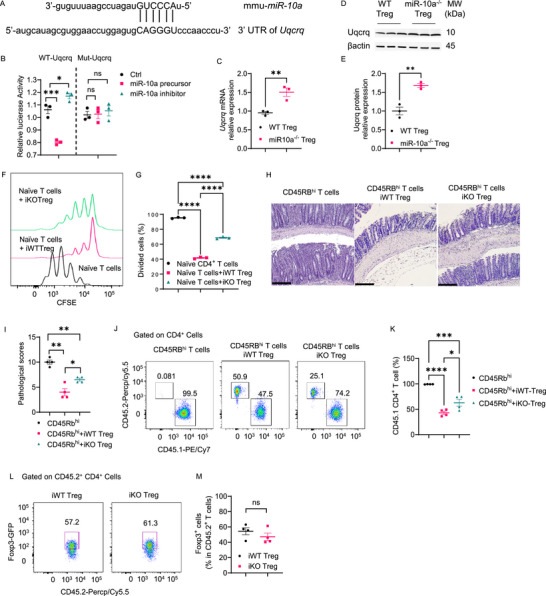
MiR‐10a modulates Treg suppressive capacity by targeting *Uqcrq*. A) Alignment of Mus musculus miR‐10a with the 3′ UTR of *Uqcrq*. B) Luciferase activity of *Uqcrq* WT and Mut 3′ UTR constructs (*n* = 3 per group) was measured. C) mRNA expression of *Uqcrq* in WT and miR‐10a‐deficient Tregs (*n* = 3 per group). D, E) Representative Western blots showing the expression of Uqcrq and β‐actin in WT and miR‐10a‐deficient Tregs (*n* = 3 per group) (D). Uqcrq protein levels were normalized to β‐actin (*n* = 3 per group) (E). F, G) CFSE‐labeled CD45.1^+^ naïve CD4^+^ T cells were co‐cultured with or without CD45.2^+^ iWT or iKO Tregs (*n* = 3 per group) in the presence of irradiated antigen‐presenting cells and soluble anti‐CD3 antibody for 60 h. Representative flow cytometry histogram plots of CFSE intensity in CD45.1^+^ CD4^+^ T cells (F). Quantification of divided CD45.1^+^ CD4^+^ T cells (G). H–M) Splenic CD45.1^+^ CD4^+^ CD45Rb^hi^ were transferred to *Rag1* ^−/−^ mice together with or without CD45.2^+^ iWT or iKO Treg cells. Recipients (*n* = 4 per group) were sacrificed 6 weeks later. Representative H&E (H) and Pathological scores (I) of recipient mice. Representative flow cytometry histogram plots (J) and quantification (K) of colonic CD45.1^+^ cells in CD4^+^ T cells. Representative flow cytometry plots (L) and quantification (M) of colonic Foxp3‐GFP^+^ in CD45.2^+^ CD4^+^ T cells. Scale bar, 100 µm. All data are presented as mean ± SEM and are one representative of two independent experiments (A–M). One‐way ANOVA with Dunnett's multiple comparison test (B), Unpaired Student's *t*‐test (C, E, M); one‐way ANOVA with Tukey's multiple comparisons test (F, K), Mann–Whitney U test (I); ns, not significant; ^*^ *p* < 0.05, ^**^ *p* < 0.01, ^***^ *p* < 0.001, ^****^ *p* < 0.0001.

To investigate whether miR‐10a directly targets genes to regulate mitochondrial function, we searched for the potential target genes of miR‐10a using miRmap. *Uqcrq*, a predicted target of miR‐10a (**Figure** **6**A), encodes the QPC subunit of complex III, which is critical in regulating mitochondrial oxidation.^[^ ^15^ ^]^ Then, we used a luciferase assay to verify if *Uqcrq* is the target of miR‐10a. We found that miR‐10a precursor inhibited, while miR‐10a inhibitor increased, luciferase activity in the vector fused with the predicted seed sequences in the 3′UTR of *Uqcrq*, but not in the vector with mutant sequences (Figure 6B). Consistently, we found that Uqcrq mRNA and protein expression were increased in miR‐10a‐deficient Tregs (Figure 6C–E). We next used the mice harboring the *Uqcrq* floxed (QPC^fl/fl^), *Foxp3* ^GFP‐CreERT2^, and *ROSA26Sor* ^CAG‐tdTomato^ alleles (QPC iKO).^[^ ^15^ ^]^ In these mice, all the Foxp3^+^ Tregs express GFP, and cells that have undergone cre‐recombinase‐mediated loss of *Uqcrq* express tdTomato. Meanwhile, we used the mice harboring the *Foxp3^GFP‐CreERT2^* and *ROSA26Sor^CAG‐tdTomato^* alleles (QPC iWT) as control mice. In QPC iWT mice, GFP marks Foxp3^+^ Tregs, and tdTomato identifies WT Foxp3^+^ Tregs after being given tamoxifen. After being given three doses of tamoxifen, splenic uqcrq‐deficient (QPC iKO) Treg and iWT Tregs were purified from QPC iKO and QPC iWT mice. QPC iKO Tregs exhibited decreased capacity in suppressing the proliferation of naïve T cells i*n vitro* (Figure 6F,G). To investigate the effect of Uqcrq on Treg function in the colitis setting, we transferred CD45.2 QPC iKO or iWT Tregs into *Rag1* ^−/−^ mice, together with CD45.1^+^ CD45Rb^hi^ T cells. *Rag1* ^−/−^ recipients were sacrificed six weeks later. *Rag1* ^−/−^ recipients of CD45Rb^hi^ T cells and QPC iKO Tregs developed more severe colitis compared to *Rag1* ^−/−^ recipients of CD45Rb^hi^ T cells and QPC iWT Tregs (Figure 6H,I). In addition, QPC iKO Tregs were less potent in suppressing CD45.1^+^ T cell proliferation in the intestine of recipient mice (Figure 6J,K). We also found that deficiency of Uqcrq did not affect Treg stability in the intestine of recipient mice after six weeks of cell transfer (Figure 6L,M).

### 2.7 MiR‐10a Deficiency in Treg Enhances Intestinal Barrier Integrity by Targeting Areg

**Figure 7:**
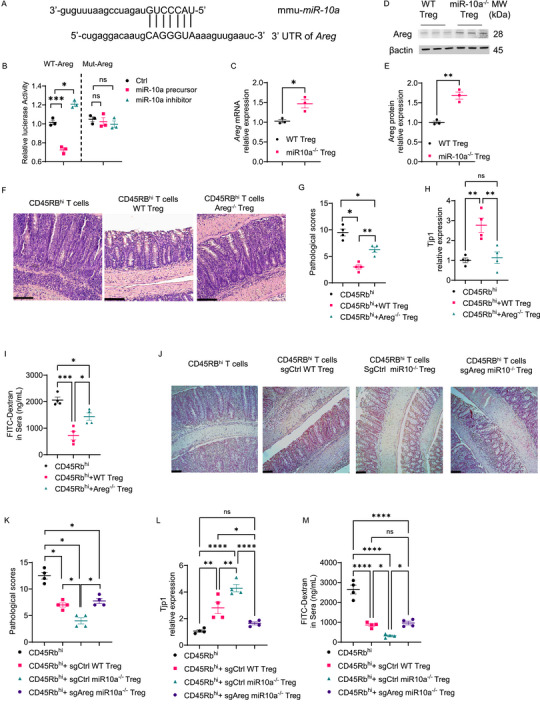
Loss of Areg, a downstream target of miR‐10, impaired Treg's capacity to promote intestinal barrier function. A) Alignment of Mus musculus miR‐10a with the 3′ UTR of *Areg*. B) Luciferase activity of *Areg* WT and Mut 3′ UTR constructs (*n* = 3 per group) was measured. C) MRNA expression of *Areg* in WT and miR‐10a‐deficient Tregs. D, E) Representative Western blots showing the expression of Areg and β‐actin in WT and miR‐10a‐deficient Tregs(*n* = 3 per group) (D). Areg protein levels were normalized to β‐actin (*n* = 3 per group) (E). F–I) Splenic CD45.1^+^ CD4^+^ CD45Rb^hi^ were transferred to *Rag1* ^−/−^ mice together with or without CD45.2^+^ WT or Areg‐deficient Tregs. Recipients (*n* = 4 per group) were sacrificed 6 weeks later. Representative H&E (F) and Pathological scores (G) of recipient mice. *Tjp1* levels in colonic epithelial cells from *Rag1* ^−/−^ recipients (H). FITC‐Dextran levels in sera were determined in *Rag1* ^−/−^ recipients (I). Scale bar, 100 µm. J–M) Splenic CD45.1^+^ CD4^+^ CD45Rb^hi^ were transferred to *Rag1* ^−/−^ mice with or without CD45.2^+^ sgCtrl WT, sgCtrl miR‐10a‐deficient, or sgAreg miR‐10a‐deficient Tregs. Recipients (*n* = 4 per group) were sacrificed 6 weeks later. Representative H&E (J) and Pathological scores (K) of recipient mice. *Tjp1* levels in colonic epithelial cells from *Rag1* ^−/−^ recipients (L). FITC‐Dextran levels in sera were determined in *Rag1* ^−/−^ recipients (M). Scale bar, 100 µm. All data are presented as mean ± SEM and are one representative of two independent experiments (A–M). One‐way ANOVA with Dunnett's multiple comparison test (B), Unpaired Student's *t*‐test (C and E); one‐way ANOVA with Tukey's multiple comparisons test (H, I, L, M), Mann–Whitney U test (G and K); ns, not significant; ^*^ *p* < 0.05, ^**^ *p* < 0.01, ^***^ *p* < 0.001, ^****^ *p* < 0.0001.

In addition to inhibiting effector T cell function to suppress intestinal inflammation, Tregs can promote intestinal epithelial repair. Interestingly, Foxp3^YFP‐cre^miR‐10a^fl/fl^ mice showed an increased intestinal barrier integrity compared with Foxp3^YFP‐cre^miR‐10a^fl/+^ mice in the DSS‐induced chronic colitis model, as demonstrated by a higher level of IEC tight junction protein 1 (Tjp1) in Foxp3^YFP‐cre^miR‐10a^fl/fl^ mice (Figure S2C, Supporting Information). It has been shown that Areg promotes intestinal healing and protects against intestinal inflammation.^[^ ^29^, ^30^ ^]^ Areg is predicted as a potential target of miR‐10a by using miRmap (**Figure** **7**A). We confirmed that miR‐10a directly targeted Areg using a luciferase assay of the vector fused with the predicted seed sequences in the 3′UTR of *Areg* (Figure 7B). Consistently, Areg mRNA and protein expression were increased in miR‐10a‐deficient Tregs compared with WT Tregs (Figure 7C–E).

Next, we transferred CD4^+^ CD45Rb^hi^ T cells together with or without Areg‐deficient or WT Tregs, isolated from Foxp3^YFP‐cre^Areg^fl/fl^ or Foxp3^YFP‐cre^Areg^fl/+^ mice, respectively, into *Rag1* ^−/−^ mice. The recipient mice were sacrificed six weeks later. *Rag1* ^−/−^ recipients of CD45Rb^hi^ T cells and Areg‐deficient Tregs developed more severe colitis compared to *Rag1* ^−/−^ recipients of CD45Rb^hi^ T cells and WT Tregs (Figure 7F,G). Although WT Tregs promoted *Tjp1* expression in IECs, Areg‐deficient Tregs failed to promote IEC expression of *Tjp1* (Figure 7H). FITC‐dextran in serum, which indicates intestinal permeability, was higher in the recipients of CD45Rb^hi^ T cells and Areg‐deficient Tregs than in the recipients of CD45Rb^hi^ T cells and WT Tregs (Figure 7I).

To further confirm that miR‐10a–deficient Tregs enhance intestinal barrier integrity through upregulation of Areg, we used nucleofection to deliver a CRISPR‐Cas9 ribonucleoprotein (RNP) complex, including Cas9 protein and single‐guide RNA (sgRNA),^[^ ^31^ ^]^ into miR‐10a–deficient Tregs to delete the *Areg* gene (sg*Areg*). We used a negative single‐guide RNA (sgCtrl) as a control. CD4⁺ CD45RB^hi^ T cells were transferred into *Rag1* ^−/−^ mice either alone or together with sgCtrl WT Tregs, sgCtrl miR‐10a–deficient Tregs, or sg*Areg* miR‐10a–deficient Tregs. We found that Areg deletion abolished the protective effects of miR‐10a deficiency on pathology score, intestinal expression of *Tjp1*, and FITC‐dextran permeability (Figure 7J–M). These data indicate that Areg contributes to the enhanced capability of miR‐10a‐deficient Tregs to promote barrier integrity.

To assess whether *Prdm1*, *Uqcrq*, and *Areg* are co‐expressed within the same Treg subsets, we visualized their expression patterns in UMAP (Figure S6A–C, Supporting Information). *Prdm1* and *Areg* are predominantly enriched in eTregs and NLT‐like Tregs (Figure S6A,C, Supporting Information), suggesting a shared functional context. In contrast, *Uqcrq* was broadly expressed across Treg populations (Figure S6B, Supporting Information), indicating a more generalized role. Additionally, we performed single‐cell co‐expression analysis using Feature Plot. As shown in Figure S6D–F (Supporting Information), eTregs exhibited a higher proportion of *Prdm1* ^+^ *Uqcrq* ^+^, *Prdm1*⁺*Areg*⁺, and *Uqcrq* ^+^ *Areg* ^+^ cells compared to cTreg and NLT‐like Tregs. These data suggest that miR‐10a targets are regulated in a context‐ and subset‐specific manner, particularly in eTregs. To investigate whether *Prdm1*, *Uqcrq*, and *Areg* affect each other in Tregs, we used nucleofection to deliver a CRISPR‐Cas9 RNP complex into WT Tregs to individually delete the *Prdm1* (sg*Prdm1*), *Uqcrq* (sg*Uqcrq*), or *Areg* (sg*Areg*). We found that loss of *Prdm1*, *Uqcrq*, or *Areg* did not alter the expression of the other two genes in Tregs (Figure S6G–J, Supporting Information).

## 3 Discussion

MiRNAs regulate gene expression at a post‐transcriptional level and mediate various human diseases, including IBD.^[^ ^32^ ^]^ Tregs are central in maintaining intestinal homeostasis by suppressing dysregulated immune responses. Several miRNAs have been reported to promote Treg differentiation, functions, and stability.^[^ ^33^ ^]^ Our findings demonstrated that miR‐10a negatively regulates the suppressive function of Tregs and the crosstalk between Tregs and IECs, without affecting Treg differentiation and stability.

Metabolic reprogramming was observed in miR‐10a‐deficient Treg cells. Pharmacological inhibition of mitochondrial ATP production impaired Treg suppressive functions in inhibiting colitis, suggesting that mitochondrial oxidative phosphorylation is a key element in controlling Treg activity. *Uqcrq*, a subunit of mitochondrial complex III, was identified as a target gene of miR‐10a. Loss of *uqcrq* decreased Treg capacity in suppressing intestinal inflammation. Although mitochondrial metabolism has been found to regulate Treg stability,^[^ ^34^ ^]^ we did not observe a difference in Treg stability between WT and miR‐10a‐deficient Treg cells. Consistently, deficiency in *uqcrq* did not affect Treg stability. This suggests a functional dichotomy wherein miR‐10a plays a critical role in modulating the suppressive capacity of Tregs without affecting their stability. One possibility is that miR‐10a‐deficient Tregs may adapt to mitochondrial metabolic changes by utilizing alternative metabolic pathways, which contribute to maintaining Treg stability. However, this metabolic flexibility may not be sufficient to sustain Treg suppressive activity, indicating that Treg stability and function are regulated by distinct molecular and metabolic pathways.

Areg, a member of the EGF family, plays a significant role in maintaining intestinal integrity and protecting against colitis.^[^ ^30^, ^35^ ^]^ Here, we found that Areg was one of the targets of miR‐10a, and Areg‐deficient Tregs failed to promote intestinal barrier repair. In addition, Treg‐specific miR‐10a‐deficient mice displayed enhanced intestinal barrier integrity compared to control mice in the chronic DSS colitis model. These findings suggest that miR‐10a restricts Treg‐mediated intestinal barrier function via directly targeting Areg.

Tregs are heterogeneous, consisting of various subsets exhibiting distinct phenotypic and functional characteristics.^[^ ^36^ ^]^ Tregs can be classified into cTreg and eTreg based on their activation status and suppressive capacity. Relative to cTregs, eTregs are activated and express higher levels of suppressive markers, thereby exerting a more potent suppressive function to control inflammation.^[^ ^37^ ^]^ The shift toward more functional subsets correlates with enhanced suppressive capacity. Single‐cell RNA sequencing revealed that miR‐10a deficiency in Treg was associated with a shift toward an eTreg phenotype. One limitation of this single‐cell dataset is the use of pooled samples per genotype, which does not allow evaluation of biological variability among individual mice. Nevertheless, this finding was validated by measuring CD44 and CD62L using flow cytometry. Blimp1 is recognized as a critical player in regulating Treg suppressive function. Our data indicate that Blimp1 is a direct target of miR‐10a, suggesting a mechanistic basis by which deficiency of miR‐10a promotes suppressive capacity in Treg cells. Given that Blimp1, jointly with IRF4, has been reported to control the differentiation and function of eTregs,^[^ ^8^ ^]^ these findings suggest that Blimp1 at least partially contributes to eTreg expansion in miR‐10a^−/−^ Tregs. Cellular metabolism plays a critical role in dictating Treg differentiation and function.^[^ ^13^ ^]^ Uqcrq, a component of mitochondrial complex III, may influence Treg fate by modulating mitochondrial metabolism.^[^ ^15^ ^]^ Although eTregs are reported to be more glycolytic than cTregs,^[^ ^37^ ^]^ mitochondrial respiration remains essential for biosynthetic and signaling functions.^[^ ^38^ ^]^ While our data suggest that Uqcrq contributes to the metabolic fitness required for Treg suppression, additional studies will be needed to determine whether Uqcrq directly drives the shift toward eTregs. Areg, an epidermal growth factor family member implicated in tissue repair and immune responses,^[^ ^30^, ^35^ ^]^ typically serves as a functional marker of eTregs and reflects their tissue‐repair capacity rather than directly regulating the cTreg and eTreg differentiation. Together, these findings suggest that miR‐10a directly controls a network of metabolic and transcriptional targets, including Blimp1, Uqcrq, and Areg, that cooperatively shape Treg subset differentiation and function.

Our current study focuses on murine models, and the clinical relevance of miR‐10a in human IBD remains to be determined. Future studies are needed to evaluate miR‐10a expression in Tregs from inflamed vs uninflamed intestinal mucosa of IBD patients, and to assess whether altered miR‐10a levels correlate with Treg subset imbalance or dysfunction. From a translational perspective, microRNA‐based approaches such as miRNA inhibitors or miRNA mimics are under active development in oncology and immune disorders.^[^ ^39^, ^40^ ^]^ While several challenges must be addressed, including efficient and selective delivery to intestinal Tregs, minimizing off‐target effects, and avoiding unintended immunological consequences due to miR‐10a expression in other immune cell types, our findings provide a conceptual framework for targeting miR‐10a in immunoregulatory pathways. Moreover, *ex vivo* modulation of miR‐10a in human Tregs could provide mechanistic and therapeutic insight into its regulatory potential. By modulating Treg‐associated metabolic and suppressive programs, miR‐10a‐based strategies may ultimately contribute to restoring immune homeostasis in IBD.

In summary, this study reveals the complex regulatory roles of miR‐10a in Treg functions, contributing to the pathogenesis of intestinal inflammation. Loss of miR‐10a promotes a shift toward an activated subset of eTregs with higher expression of Blimp1. Additionally, the deficiency of miR‐10a increased mitochondrial oxidative phosphorylation by targeting *Uqcrq* in Tregs, which affects Treg suppressive function without influencing Treg stability. Furthermore, miR‐10a restricted Treg‐mediated intestinal barrier function by targeting Areg. These findings suggest the roles of miR‐10a in regulating both Treg suppressive capacity and Treg‐mediated intestinal barrier function.

## 4 Experimental Section

### Mice

C57BL/6J wild‐type (WT) mice, B6.129(Cg)‐Foxp3^4(YFP/icre)Ayr^/J (*Foxp3* ^YFP‐cre^) mice, and B6.129‐Prdm1^1Clme^/J (*Prdm* ^fl/fl^) mice were purchased from Jackson Lab. *MiR‐10a* ^fl/fl^ mice were generated as described previously. *Foxp3* ^YFP‐cre^ *Areg^f^* ^l/fl^ and *Foxp3* ^YFP‐cre^ *Areg* ^fl/+^ mice were kindly provided by Dr. Karen Ridge of Northwestern University. QPC^fl/fl^ mice, QPC iWT, and iKO mice were kindly provided by Dr. Navdeep Chandel of Northwestern University. 40 mg mL^−1^ tamoxifen was administered to QPC iWT and iKO mice three times in 8‐week‐old mice, and splenic Tregs were collected six weeks after the last treatment of tamoxifen. All the mice were maintained in the specific pathogen‐free animal facility of the University of Texas Medical Branch or the Center for Comparative Medicine at Northwestern University. All animal experiments were approved by the Institutional Animal Care and Use Committee (IACUC) of the University of Texas Medical Branch (2004044) or the Center for Comparative Medicine at Northwestern University (IS00027174). All the mice used in this study were sex‐matched and 6–12 weeks of age.

### Mouse Model

### Mouse Model—DSS‐Induced Chronic Colitis

Foxp3^YFP‐cre^miR‐10a^fl/fl^ mice and Foxp3^YFP‐cre^miR‐10a^fl/+^ littermates were administered with a 2% DSS (w/v) in the drinking water for seven days, followed by 7 days of regular drinking water, and repeated three times.

### Mouse Model—CD4^+^ CD45Rb^hi^ T Cell‐Transfer Colitis

Splenic CD45.1^+^ CD4^+^ CD45Rb^hi^ (100K cells per mouse) together with or without CD45.2^+^ CD4^+^ Foxp3^+^ T cells (50K cells per mouse) were transferred to *Rag1* ^−/−^ mice by tail intravenous injection.

### Hematoxylin and Eosin Staining and Histopathological Evaluation

Mouse colons were Swiss‐rolled, fixed, dehydrated, cleared, and embedded in paraffin. Paraffin‐embedded samples were then cut into 5‐µm sections. After deparaffinization and hydration, the slides were stained with hematoxylin and eosin.

The stained slides were imaged using a Leica DM1000, and the histopathological evaluation was conducted based on specific criteria tailored to each colitis model. For DSS‐induced chronic colitis, two parameters were used: cell infiltration (scored as 0: normal; 1: mild in mucosa; 2: moderate in mucosa; 3: marked in mucosa; 4: moderate/severe in mucosa and submucosa; 5: transmural) and architecture damage (scored as 0: no erosion 0; 1: focal erosion; 2: focal ulceration; 3: extended ulcerations 0–25%; 4: 25%–50%; 5: 50%–75%; 6: >75%). For CD4^+^ CD45Rb^hi^ T cell‐transfer colitis: the pathology scoring was performed using six parameters: lamina propria inflammation (scored as 0: normal; 1: mild; 2: moderate; 3: severe), goblet cell loss (scored as 0: normal; 1: mild; 2: moderate; 3: severe), abnormal crypt (scored as 0: normal; 1: hyperplastic; 2: disorganization; 3: crypt loss), crypt abscesses (scored as 0: absent; 1: present), mucosal erosion and ulceration (scored as 0: normal; 1: mild; 2: moderate; 3: severe), and submucosal change (scored as 0: none; 1: submucosa; 2: transmural).

### Isolation of CD4^+^ CD45Rb^hi^ T Cells, Naïve CD4^+^ T Cells, and Tregs

Mouse spleens were ground, and red blood cells were lysed. Splenic CD4^+^ T cells were then isolated using anti‐mouse CD4 magnetic particles. Cells were stained with anti‐mouse CD4, anti‐mouse CD45b, and PI for the purification of CD4^+^ CD45Rb^hi^ cells; anti‐mouse CD4, anti‐mouse CD62L, and PI for the isolation of naïve CD4^+^ T cells; or anti‐mouse CD4 antibody and PI for the isolation of Treg cells. Cell sorting was performed on a Sony SH800s sorter. CD4^+^ CD45Rb^hi^ cells were gated on PI^−^ CD4^+^ CD45Rb^hi^, naïve CD4^+^ T cells were gated on PI^−^ CD4^+^ CD62L^−^, and Tregs were gated on PI^−^ CD4^+^ Foxp3^+^ (YFP^+^ or GFP^+^ tdTamato^+^).

### Pretreatment in Tregs

Treg cells were pretreated with oligomycin (3 nm) or MitoQ (100 nm) for 2 h. Cells were then washed with 1 × PBS twice.

### Flow Cytometry

Cells were washed with 1× PBS and incubated with anti‐CD16/32 for 5 min. Subsequently, cells were stained with live dye using LIVE/DEAD Fixable Near‐IR Dead Cell Stain Kit, followed by staining with anti‐CD45.1, anti‐CD45.2, and anti‐CD4. After permeabilization using Foxp3/Transcription Factor Fixation/Permeabilization set, cells were stained with anti‐IFN‐γ, anti‐IL‐17A, and anti‐Foxp3.

For staining mitochondrial mass and mitochondrial membrane potential, Foxp3‐YFP^+^ Tregs were washed with 1× PBS and stained with live dye using LIVE/DEAD Fixable Near‐IR Dead Cell Stain Kit. Cells were then incubated with MitoTracer Deep Red FM and MitoTracer Red CMXRos at 37 °C for 15 min.

### Western Blot

Tregs were lysed by RIPA lysis buffer supplemented with protease, phosphatase inhibitors, and PMSF. Protein concentrations were quantified by assay using BCA kits, and equal amounts of protein (5–15 µg) were separated on Bio‐Rad Mini‐PROTEIN TGX precast gels and transferred onto PVDF membranes using a semi‐dry transfer system (Bio‐Rad). Membranes were blocked with 5% non‐fat milk in TBS with 0.5% (v/v) Tween 20 (TBST) for 1 h at room temperature and then incubated with primary antibodies in TBST overnight at 4 °C. After washing, membranes were incubated with HRP‐conjugated secondary antibodies for 1 h at room temperature. Protein bands were visualized using ECL reagents (Bio‐Rad) and imaged with a ChemiDoc imaging system. For reprobing, membranes were stripped using Restore PLUS Western Blot Stripping Buffer (Thermo Fisher Scientific), followed by re‐blocking and incubation with the next primary antibody. Band intensities were quantified using ImageJ and normalized to β‐actin.

### In Vitro T Cell Suppression Assay

Splenic CD45.1^+^ naïve CD4^+^ T cells were labeled with CFSE and then co‐cultured with or without CD45.2^+^ Tregs in the presence of irradiated splenic antigen‐presenting cells and soluble anti‐CD3 (5 µg mL^−1^) for 60 h. CFSE intensity in CD45.1^+^ CD4^+^ T cells was analyzed by flow cytometry.

### Intestinal Lamina Propria Cell Isolation

Mouse colons were opened longitudinally and cut into 1‐cm pieces. After removing intestinal epithelial cells by incubation with EDTA for 30 min, tissues were digested by Collagenase IV and DNAse in the gentleMACS tubes on the gentleMACSTM Dissociator, following the program ″37C_m_LPDK_1″, repeated twice. All the cells were collected by passing through a 100‐µm cell strainer, and intestinal lamina propria cells were purified using a 40%/70% Percoll gradient.

### Foxp3 Methylation Panel

Methylation in different sites of Foxp3 was analyzed by NextGene Sequencing (Epigendx), and the percentages of methylation were calculated in Bismark by dividing the number of methylated reads by the total number of reads.

### Dual Luciferase Assay

RAW 264.7 cells were seeded in 24‐well plates and transfected with Psi‐CHECK‐2 plasmids containing either the WT or mutated 3′UTR of Prdm1/Uqcrq/Areg using Lipofectamine 3000 in Opti‐MEM. After 24 h, cells were lysed, and luciferase activity was measured using the Dual‐Luciferase Reporter Assay System (Promega). Renilla luciferase activity was normalized to Firefly luciferase activity to account for transfection efficiency.

### Cellular Metabolic Measurement

WT and miR‐10a‐deficient Tregs (200K/well) were seeded in the poly‐lysine‐coated XF96 cell culture microplate. The Seahorse XF Cell Mito Stress Test Kit was used to determine the key parameters of mitochondrial respiration by measuring the oxygen consumption rate (OCR) using the Seahorse XF96 Analyzer.

### FITC‐Dextran Assay

Mice were fasted for 6 h and then treated with FITC‐Dextran solution (4 kDa, 12 mg per mouse) by oral gavage. After 4 h, sera were collected and centrifuged at 15 000 g for 15 min. Serum FITC‐Dextran levels were determined with 490 excitation/520 emission by BioTek Synergy HTX Multimode Reader.

### Quantitative Real‐time PCR

Total RNA was extracted from cells using TRIzol reagent and reverse‐transcribed into complementary DNA (cDNA) with the iScript cDNA Synthesis Kit (Bio‐Rad). Quantitative real‐time PCR was conducted using SYBR Green Master Mix (Bio‐Rad) on a Bio‐Rad real‐time PCR system. Gene expression levels were normalized to βactin as an internal control and analyzed using the 2^−ΔΔCt^ method. The primers used are shown in Table S1 (Supporting Information).

### ATP Assay

Intracellular ATP levels in WT and miR‐10a‐deficient Tregs were measured using the CellTiter‐Glo Luminescent Cell Viability Assay (Promega). 3×10⁴ Tregs in 100 µL of PBS were seeded into a white 96‐well plate, and 100 µL of CellTiter‐Glo reagent was added to the plate. After shaking the plates on an orbital shaker, incubate the plate for 10 min at room temperature. Luminescence was determined using a plate reader (BioTek Synergy).

### CRISPR‐Cas9 RNP Nucleofection

Cas9 (Alt‐R S.p. Cas9 Nuclease V3, Integrated DNA Technologies) and sgRNAs (Synthego) were combined and incubated at room temperature for 10 min. For each target, two sgRNAs were used to increase knockout efficiency. Non‐targeting Negative controls sgRNA #1 and # 2 (Synthego) were used. Tregs (5 million per sample), suspended in 20 µL of P3 nucleofector solution (P3 Primary Cell 96‐well Nucleofector Kit), were mixed with RNP, followed by nucleofection using the 4D‐Nucleofector X Unit (Lonza) with program DN100. The sgRNA sequences used are shown in Table S2 (Supporting Information).

### Single‐RNA Sequencing

Live splenic Foxp3^+^ Tregs were sorted from WT and Treg‐specific miR‐10a‐deficient mice by gating on PI^−^ CD4^+^ Foxp3^+^. Each condition (WT Tregs and miR‐10a‐deficient Tregs) was generated by pooling cells from three individual mice, and single‐cell capture and library preparation were performed as one sample per group. Single‐cell RNA libraries were constructed using the Chromium Next GEM Single Cell 5′ Reagent Kit v2 (10x Genomics) following the manufacturer's protocol. Briefly, single‐cell suspensions were loaded onto a Chromium Controller to generate single‐cell gel bead‐in‐emulsions (GEMs), followed by reverse transcription and cDNA amplification. Libraries were prepared using the 10× Genomics Library Kit and quantified using an Agilent Bioanalyzer with a High Sensitivity DNA Chip. Dual indexing was performed using the Dual Index Kit TT Set A. Sequencing was performed on an Illumina NovaSeq 6000 platform with a paired‐end 150 bp configuration. A total of 2 premade libraries were sequenced, generating 500 million reads per library (∼300 Gb total). Sequencing was performed on a partial NovaSeq lane (∼300 Gb) to ensure full data coverage. Raw sequencing data were processed using the Cell Ranger pipeline (10x Genomics) to generate gene expression matrices, which were subsequently analyzed using Loupe Browser (10x Genomics) for visualization and exploration. Cells were filtered using the following criteria: log2(UMIs) between 10 and 14, number of detected genes between 1000 and 3500, and mitochondrial UMIs <5%. After QC filtering, 17,567 cells were retained, including 8264 cells from WT Tregs and 9303 cells from miR‐10a‐deficient Tregs. Graph‐based clustering of single‐cell RNA‐seq data was performed using the 10x Genomics Loupe Browser, which employs a default Louvain‐based graph clustering algorithm with the clustering resolution internally determined and not user‐accessible. UMAP visualization was generated with number of neighbors = 15 and minimum distance = 0.1.

### Bulk‐RNA Sequencing

Total RNA was extracted from WT and miR‐10a‐deficient Tregs. RNA integrity was assessed using an Agilent Bioanalyzer with an RNA 6000 Nano Chip, and only samples with an RNA integrity number (RIN) > 7.0 were used for downstream analysis. mRNA libraries were prepared using poly(A) enrichment, and sequencing was conducted using NovaSeq PE150, generating 6 Gb of raw data per sample. Quantification and analysis were performed to ensure data integrity. Reads were aligned to the reference genome (GRCh38), and gene expression levels were quantified with featureCounts (v2.0.1). Correlation analysis was conducted for biological replicates, and differential expression analysis between sample groups was performed using DESeq2. Enrichment network and Protein Complex Network MCODE analysis were performed using Metascape.^[^ ^41^ ^]^

### Statistical Analysis

The data were statistically analyzed using GraphPad Prism 10. Data are presented as mean ± SEM, with the sample size (*n*) indicated in each figure legend. A *p*‐value < 0.05 was considered statistically significant. Data preprocessing (e.g., normalization) where appropriate. Comparisons between two groups were performed using a two‐tailed Student's t‐test (for normally distributed data) or a Mann–Whitney U test (for non‐normally distributed data). For comparisons among three or more groups, one‐way or two‐way ANOVA was used, followed by Tukey's or Sidak's post‐hoc test as appropriate.

### Data Availability Statement

All sequencing data generated in this study have been deposited in the NCBI Sequence Read Archive (SRA) and are publicly available under the following accession numbers: PRJNA1222867 (bulk RNA‐seq) and PRJNA1223039 (single‐cell RNA‐seq).

## Conflict of Interest

The authors declare no conflict of interest.

## Author Contributions

W.Y. and T.Y. contributed equally to this work. W.Y., T.Y., and Y.C. did Conceptualization. W.Y., T.Y., W.C., and Y.C. did methodology. W.Y., T.Y., H.Y., S.Y., R.M., E.S., K.R., W.C., N. C., and Y.C. did investigation. K.R., N.C. and Y.C. did resources. W.Y., T.Y., and Y.C. wrote the original draft. W.Y., T.Y., and Y.C. wrote the review and did editing with input from all other authors. W.Y. and Y.C. supervised the work. W.Y., K.R., and Y.C. did funding acquisition.

## Supporting information



Supporting Information

## Data Availability

The data that support the findings will be available in the Sequence Read Archive (SRA) at https://www.ncbi.nlm.nih.gov/bioproject/PRJNA1222867; https://www.ncbi.nlm.nih.gov/bioproject/PRJNA1223039 following an embargo from the date of publication to allow for commercialization of research findings.
