# Supplementary material for: Intrinsic MicroRNA‐10a Restricts Regulatory T Cell Suppressive Function and Intestinal Repair by Coordinating Transcriptional, Metabolic, and Epithelial Repair Pathways
Source: Adv Sci (Weinh). 2025 Nov 3;13(4):e09953. doi: 10.1002/advs.202509953 (PMC12822379; doi:10.1002/advs.202509953)
Supplement: Supplementary file 1 — Supporting Information [file ADVS-13-e09953-s001.pdf]

Intrinsic MicroRNA-10a Restricts Regulatory T Cell Suppressive Function and Intestinal Repair by Coordinating Transcriptional, Metabolic, and Epithelial Repair Pathways

Wenjing Yang, Tianming Yu, et al.

Supplementary Figures

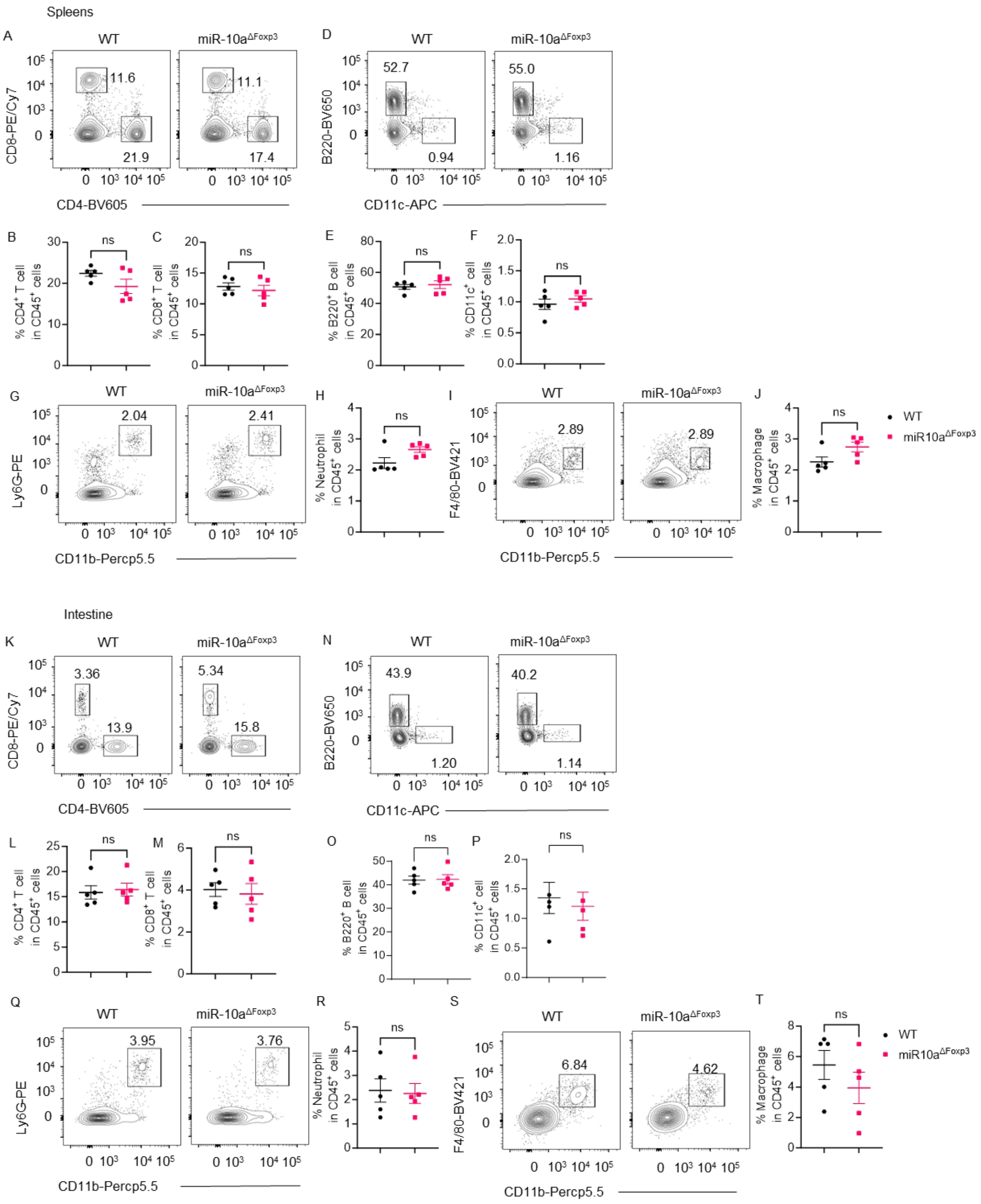

Supplementary Figure 1. Immune cell profiles in the spleens and intestines from Foxp3$^{YFP\text{-}cre}$miR-10a$^{fl/fl}$ mice and WT Foxp3$^{YFP\text{-}cre}$miR-10a$^{fl/+}$ mice.

(A-I) Representative flow cytometry plots (A, D, G, and I) and quantification (B, C, E, F, H, and J) of immune cells in the spleens from Foxp3$^{YFP\text{-}cre}$miR-10a$^{fl/fl}$ mice and WT Foxp3$^{YFP\text{-}cre}$miR-10a$^{fl/+}$ mice (5/group).

(K-T) Representative flow cytometry plots (K, N, Q, and S) and quantification (L, M, O, P, R, and T) of immune cells in the intestines from Foxp3$^{YFP\text{-}cre}$miR-10a$^{fl/fl}$ mice and WT Foxp3$^{YFP\text{-}cre}$miR-10a$^{fl/+}$ mice (5/group).

All data are presented as mean ± SEM and are one representative of two independent experiments (E-J). Unpaired Student's *t*-test; ns, not significant.

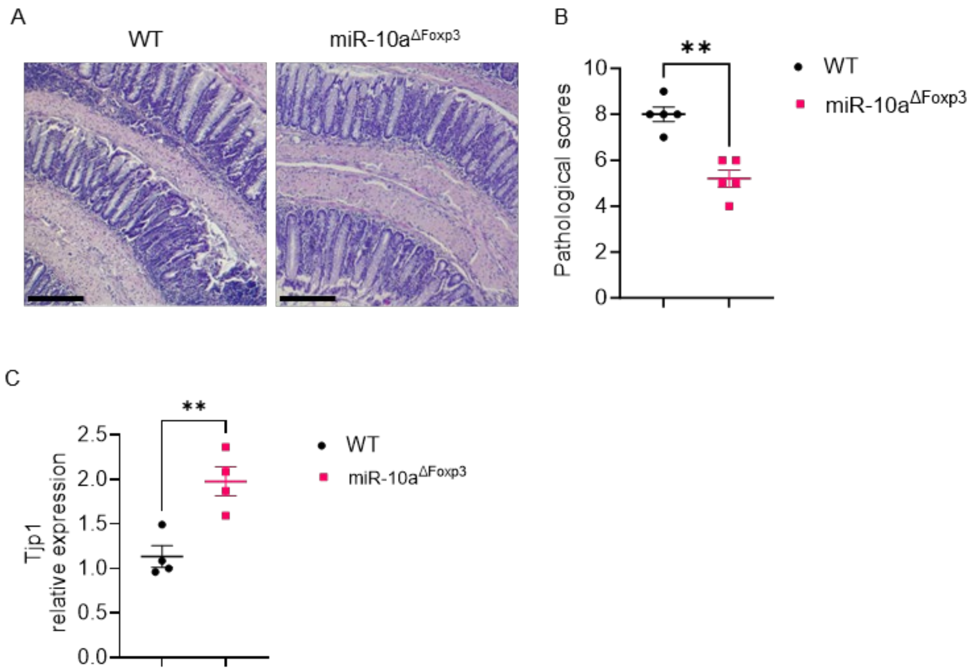

Supplementary Figure 2. Treg-specific miR-10a-deficient mice are resistant to chronic DSS insults.

WT Foxp3$^{YFP-cre}$miR-10a$^{fl/+}$ mice (5/group) and Foxp3$^{YFP-cre}$miR-10a$^{fl/fl}$ mice (5/group) were administered with 3 cycles of 2% of DSS in drinking water for 7 days followed by 7 days of regular drinking water. (A) Representative H&E and (B) and Pathological scores of two groups of mice. (c) *Tjp1* levels in colonic epithelial cells from *Rag1*$^{-/-}$ recipients. Scale bar, 100 µm.

All data are presented as mean ± SEM and are one representative of two independent experiments (A-C). Mann–Whitney U test (B), unpaired Student's *t*-test (C); ns, not significant; ***p* < 0.01.

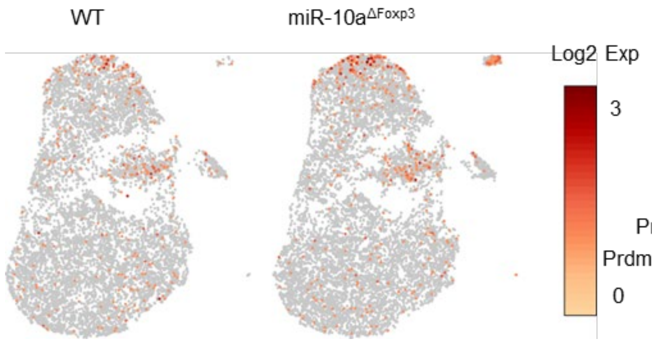

	WT	miR-10a$^{\Delta Foxp3}$
Total cell number	8264	9303
Prdm1-expressing cell number	293	442
Prdm1-expressing cell percentage	3.55%	4.75%

Supplementary Figure 3. Prdm1 expression in WT and miR-10a-deficient Treg.

UMAP visualization of single-cell RNA-sequencing data showing *Prdm1* expression in WT and miR10a-deficient Tregs.

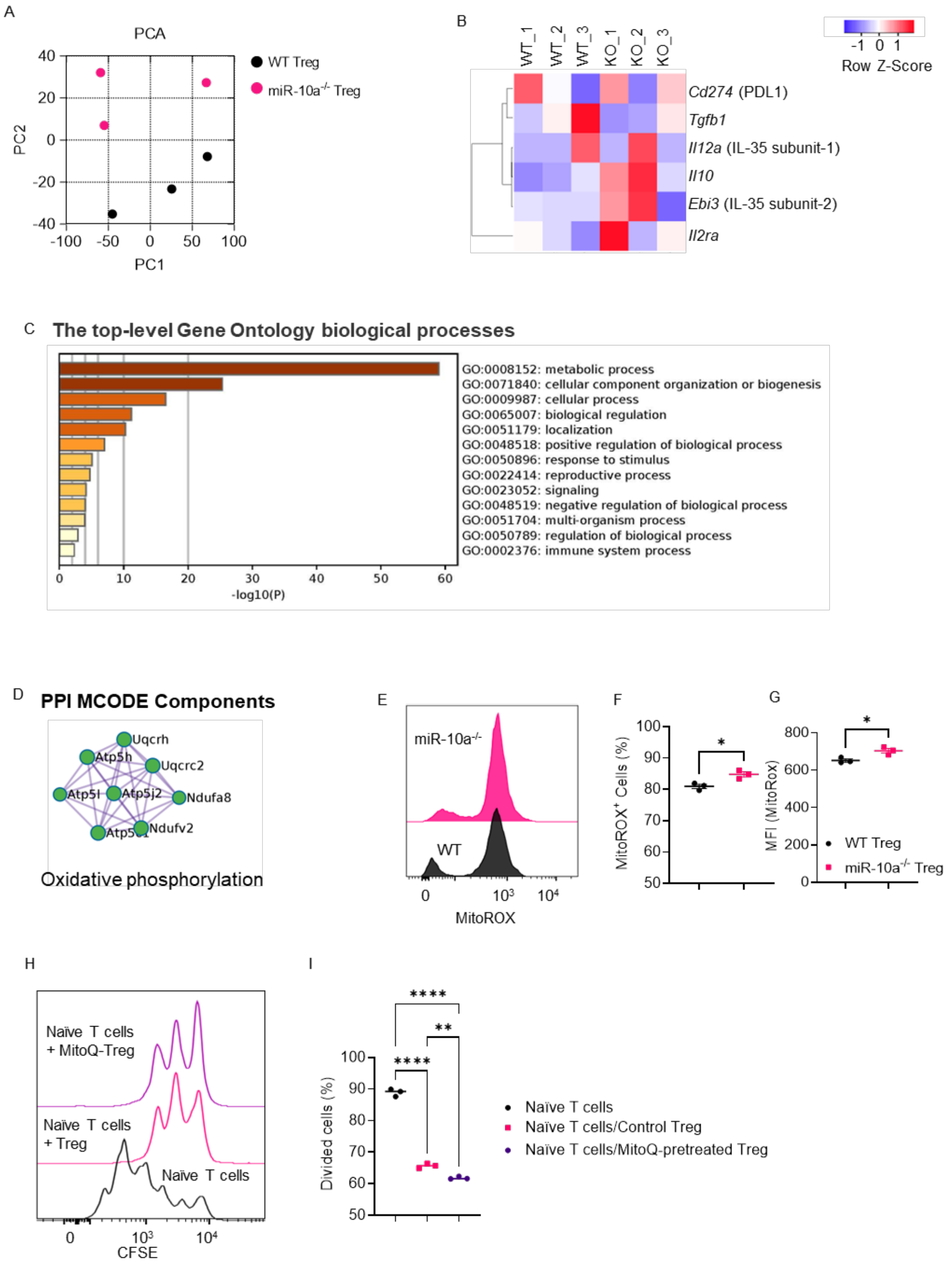

Supplementary Figure 4. Deficiency of miR-10a affects oxidative phosphorylation and mitochondrial reactive oxygen species in Tregs.

(A-C) RNA sequencing was performed in WT and miR-10a-deficient Tregs (n = 3/group). Principal Component Analysis (PCA) (A), the heatmap of selected genes (B), the enrichment analysis of top-level gene ontology (GO) biological processes derived from the differentially expressed genes (C), and the PPI network constructed from the differentially expressed genes, with the MCODE algorithm applied to identify densely connected modules (D).

(E-G) Representative flow cytometry histogram plots (E), percentages (F), and Mean Fluorescence Intensity (MFI) (G) of MitoROX.

(H-I) CFSE-labeled CD45.1$^+$ naïve CD4$^+$ T cells were co-cultured with or without CD45.2$^+$ control or MitoQ-pretreated Tregs (2 hours of pretreatment, n = 3/group) in the presence of irradiated antigen-presenting cells and soluble anti-CD3 antibody for 60 hours. Representative flow cytometry histogram plots of CFSE intensity in CD45.1$^+$ CD4$^+$ T cells (H). Quantification of divided CD45.1$^+$ CD4$^+$ T cells (I).

All data are presented as mean ± SEM and are one representative of two independent experiments (E-I). Unpaired Student's *t*-test (F and G), one-way ANOVA with Tukey's multiple comparisons test (I); *$p < 0.05$, **$p < 0.01$, ****$p < 0.0001$.

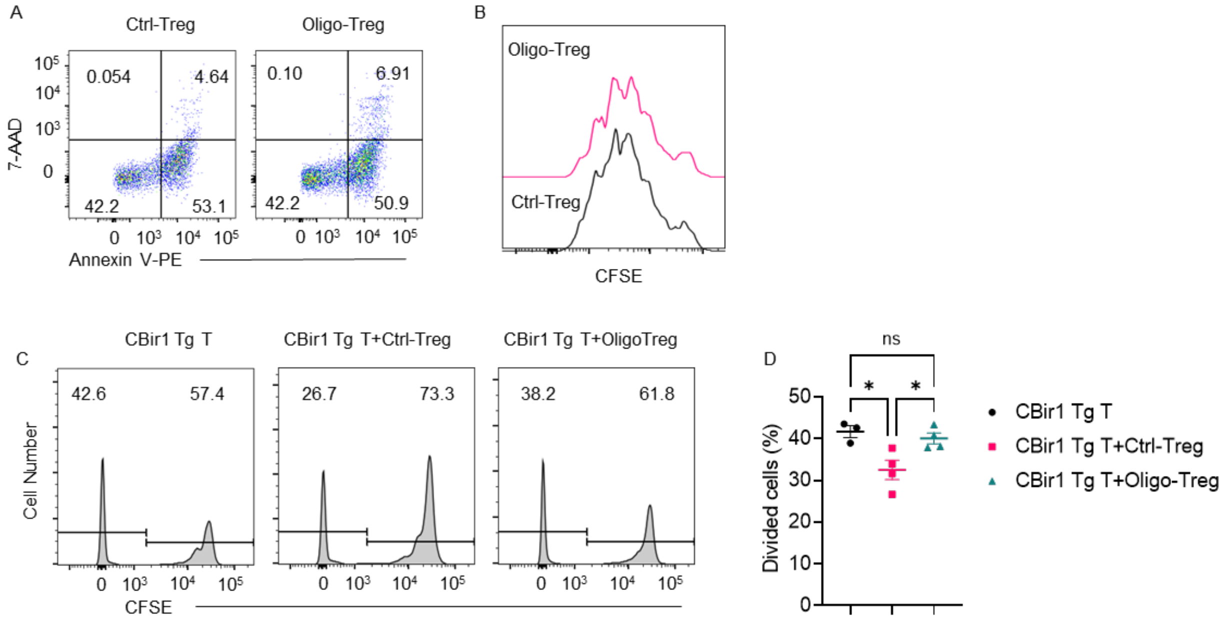

Supplementary Figure 5. Pretreatment of oligomycin at 3 nM impairs Treg suppression of CBir1 transgenic T cell proliferation.

(A-B) Tregs were pretreated with 3 nM oligomycin for 2 hours and then cultured in the presence of anti-CD3/CD28 dynabeads for 60 hours. The representative flow cytometry plots of 7-AAD and Annexin V in control Tregs and oligomycin-pretreated Tregs (A). The representative flow cytometry histogram plots of CFSE intensity in control Tregs and oligomycin-pretreated Tregs (B).

(C-D) CFSE-labeled CBir1 Tg $CD4^+$ were transferred to *Rag1*$^{-/-}$ mice together with or without WT/miR-10a-deficient Tregs (n = 4/group). Representative flow cytometry plots of CFSE intensity in TCR $V\beta8.3^+$ $CD4^+$ T cells. (C). Quantification of CFSE intensity in TCR $V\beta8.3^+$ $CD4^+$ T cells (D).

All data are presented as mean ± SEM and are one representative of two independent experiments (A-H). One-way ANOVA with Tukey's multiple comparisons test (D); ns, not significant; $*p < 0.05$.

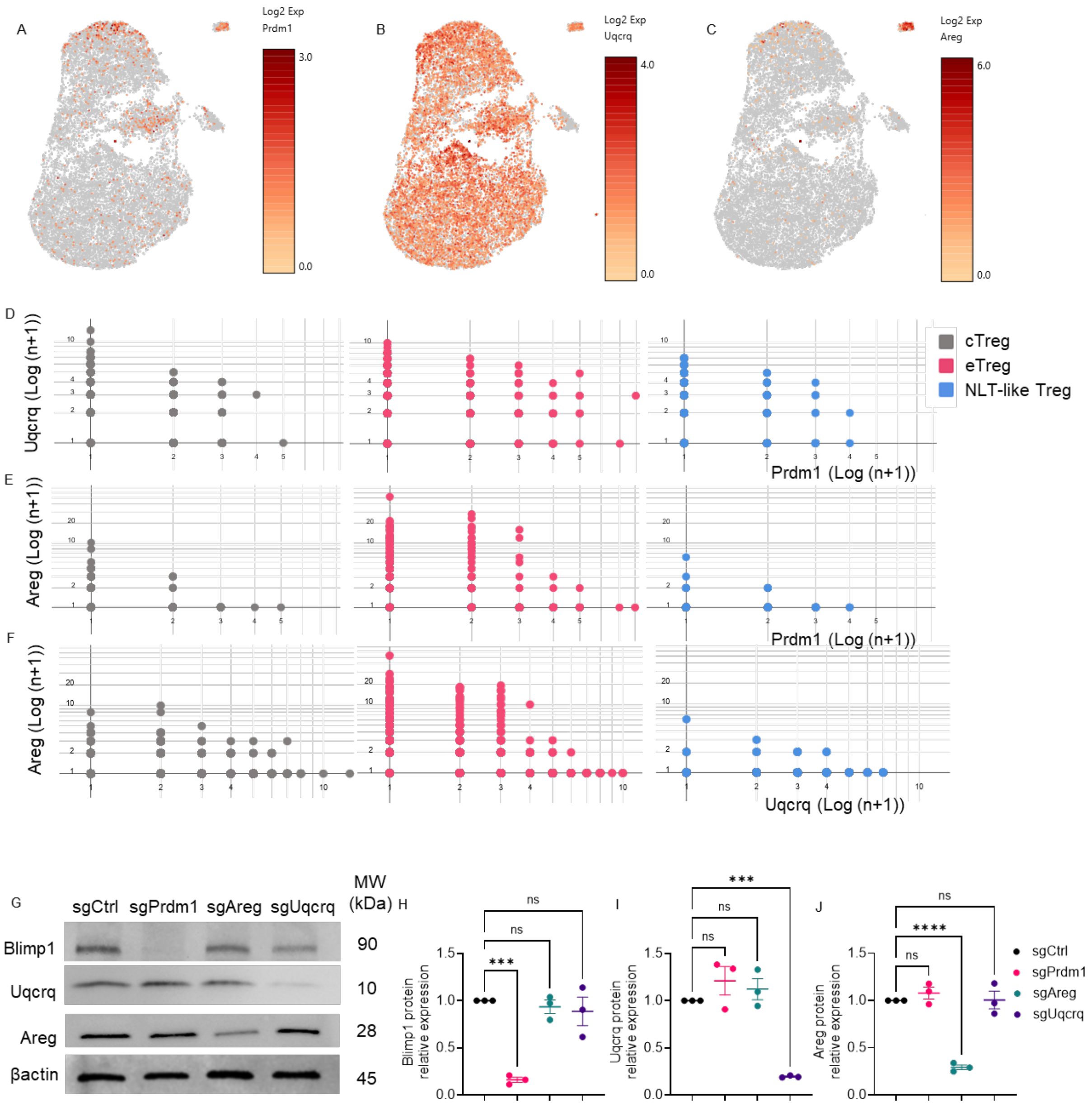

Supplementary Figure 6. The relationship between Blimp1, Uqcrq, and Areg in Tregs.

(a-c) UMAP visualization of single-cell RNA-sequencing data showing *Prdm1* (a), *Uqcrq* (b), *Areg* (c) expression in Tregs.

(d-f) Feature Plots of *Prdm1* vs *Uqcrq* (d), *Prdm1* vs *Areg* (e), and *Uqcrq* vs *Areg* (f).

(g-j) Blimp1 (g, h), Uqcrq (g, i), Areg (g, j) protein levels relative to βactin in sgctrl, sg*Prdm1*, sg*Areg*, and sg*Uqcrq* Tregs.

All data are presented as mean ± SEM and are one representative of two independent experiments (g-j). One-way ANOVA with Dunnett's multiple comparisons test (d); ns, not significant; *$p < 0.05$.

Supplementary Table 1. The primers for quantitative real-time PCR

Genes	Forword	Reverse
βactin	CATTGCTGACAGGATGCAGAAGG	TGCTGGAAGGTGGACAGTGAGG
Prdm1	AAGACGTTCGGTCAGCTCTCCA	CTGGCACTCATGTGGCTTCTCT
Uqcrq	CGCCTTCCCAAGCTATTTCAGC	CGACTGCTCAAACTCCTGGTTG
Areg	GCAGATACATCGAGAACCTGGAG	CCTTGTCATCCTCGCTGTGAGT
Klrg1	CGAGGAATGGTAGCCACTGTTAC	CCGATCCAGTAAAAGTCCTGACC
Icos	GCAGCTTTCGTTGTGGTACTCC	TGTGTTGACTGCCGCCATGAAC
Ctla4	GTACCTCTGCAAGGTGGAACTC	CCAAAGGAGGAAGTCAGAATCCG

Supplementary Table 2. The sgRNA sequencing

Genes	SgRNA #1	SgRNA #2
Prdm1	GACGAGAGUAGUCCCCCCAG	CGGAUAGGAUAAACCACCCG
Uqcrq	GUCUACCUGAUCUACACAUG	GCGCCAGGUUCCCAAACUCG
Areg	AGGGGACUACGACUACUCAG	UGCCGAUGCCAAUAGCUGCG
